# Anti-Neurotoxins from *Micrurus mipartitus* in the Development of Coral Snake Antivenoms

**DOI:** 10.3390/toxins14040265

**Published:** 2022-04-09

**Authors:** Ana Cardona-Ruda, Paola Rey-Suárez, Vitelbina Núñez

**Affiliations:** 1 Grupo de Investigación en Toxinología, Alternativas Terapéuticas y Alimentarias, Facultad de Ciencias Farmacéuticas y Alimentarias, Universidad de Antioquia, Medellin 1226, Colombia; ana.cardona3@udea.edu.co (A.C.-R.); jessica.rey@udea.edu.co (P.R.-S.); 2 Centro de Investigación en Recursos Naturales y Sustentabilidad, Universidad Bernardo O’Higgins, Santiago 8320000, Chile; 3 Escuela de Microbiología, Universidad de Antioquia, Medellin 1226, Colombia

**Keywords:** anti-neurotoxins, *Micrurus mipartitus*, coral snake antivenoms, three-finger toxins, phospholipase A_2_

## Abstract

In Colombia, the genus *Micrurus* includes 30 species, of which *M. mipartitus* and *M. dumerilii* are the most widely distributed. *Micrurus* causes less than 3% of the approximately 5000 cases of snakebite per year. The elapid envenomation caused by the snakes from the *Micrurus* genus, are characterized by the severity of their clinical manifestations, due to the venom neurotoxic components such as three-finger toxins (3FTx) and phospholipases (PLA_2_). The treatment for snakebites is the administration of specific antivenoms, however, some of them have limitations in their neutralizing ability. A strategy proposed to improve antivenoms is to produce antibodies against the main components of the venom. The aim of this work was to produce an antivenom, using an immunization protocol including the main 3FTx and PLA_2_ responsible for *M. mipartitus* lethality. The antibody titers were determined by ELISA in rabbits’ serum. The immunized animals elicited a response against toxins and whole venom. The Immunoglobulin G (IgGs) obtained were able to neutralize the lethal effect of their homologous toxins. A combination of antivenom from *M. mipartitus* with antitoxins improved their neutralizing ability. In the same way, a mixture of anti 3FTx and PLA_2_ protected the mice from a 1.5 median lethal dose (LD_50_) of *M. mipartitus* venom. The results showed that this might be a way to improve antibody titers specificity against the relevant toxins in *M. mipartitus* venom and indicated that there is a possibility to develop and use recombinant 3FTx and PLA_2_ toxins as immunogens to produce antivenoms. Additionally, this represents an alternative to reduce the amount of venom used in anti-coral antivenom production.

## 1. Introduction

The Elapidae family includes around 389 species worldwide; 120 of these species and subspecies are found in America, belonging to the genera *Micruroides*, *Micrurus* and *Hydrophis*. Their distribution ranges from the southern United States to northern Argentina [1].

In Colombia, the genus *Micrurus* includes 30 species, but *Micrurus mipartitus* and *M. dumerilii* are the most widely distributed; coral snakes cause less snakebite cases than the Viperidae family, they are non-aggressive snakes and only bite when they are handled or when humans come into direct contact with them. They cause less than 3% of the approximately 5000 cases of snake bite per year [2]. Although elapid envenomation caused by the snakes from the genus *Micrurus* is not the most common form of snakebite, they are noticeable by the severity of their accidents. The species of the *Micrurus* genus induce neurotoxic effects due to the presence of 3-finger toxins (3FTxs) and phospholipases A_2_ (PLA_2_) [3,4,5,6].

**Figure 1:**
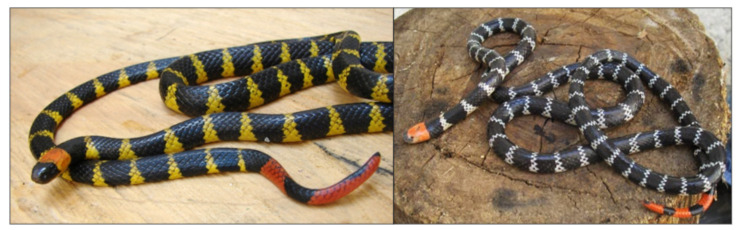
*Micrurus mipartitus* snake. Orange nuchal band and red terminal rings are evident. Source: Serpentarium University of Antioquia.

*M. mipartitus*, named “rabo de ají”, “rabo de candela”, “gargantilla”, “red-tailed coral snake” or “chocho head”, is a species with a pattern of 34 to 84 rings alternated between black and white or yellow; there are one to nine rings on the tail, alternated with red. Its head is black with an orange or red nuchal ring, wider than those of the rest of the body (Figure 1) [7,8]. The lethal dose of *M. mipartitus* has been reported as 9 µg/mouse [9].

The proteome of *M. mipartitus* venom demonstrates that three-finger toxins (3FTx) represent 61% and phospholipases A_2_ (PLA_2_) 28% of the venom weight [10]. Mipartoxin-I was identified as the 3FTx most abundant and that has a lethal effect [11]. Similarly, a lethal PLA_2_ with neurotoxic effects was identified and named MmipPLA_2_ [12].

Snakebite envenoming was classified by the World Health Organization (WHO) as a neglected tropical disease, and actions focused on improving the production and quality of antivenoms are necessary [13]. With the introduction of tools such as proteomics in venom analysis (venomics), it has been possible to identify the toxins responsible for the lethality of snakebites from species such as *M. mipartitus* [11]. These toxins could be used in developing antivenoms with greater recognition and neutralization than the antivenoms developed with the whole venom, especially in venoms which main components are low molecular weight proteins, including PLA_2_ and 3FTxs, as observed in *Micrurus* venoms. For this reason, the aim of this work was to evaluate an antivenom prepared with antitoxin antibodies in their ability to neutralize the lethal effect of *M. mipartitus* venom.

## 2. Results

### 2.1. Production and Evaluation of Hyper Immune Sera

**Figure 2:**
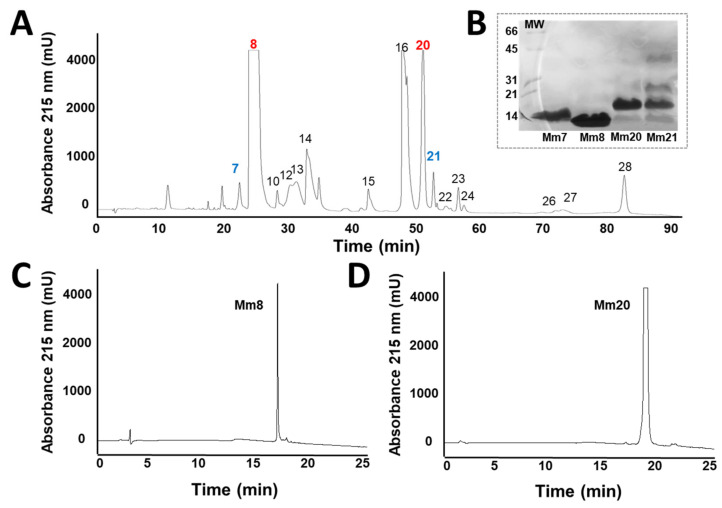
(**A**): Elution profile of *Micrurus mipartitus* venom proteins by RP-HPLC. Two mg of venom was fractionated on a C18 column, as described in the materials and methods. The numbers show the fractions selected for evaluation of the lethal effect. Numbers in blue indicate the fractions that they showed a lethal effect in mice and numbers in red indicate the most lethal and abundant fractions and those used as immunogens. (**B**): Lethal fractions were analyzed by 15% gradient SDS-PAGE under non-reducing conditions. Mm7: fraction 7, Mm8: fraction 8, Mm20: fraction 20 and Mm21: fraction 21, molecular mass markers indicated in the left (kDa). (**C**): Mm8 (50 µg) and (**D**): Mm20 (100 µg) toxin purity profile by RP-HPLC, as described in the materials and methods.

The *M. mipartitus* venom showed 28 fractions (Figure 2), the eight (Mm8) and twenty (Mm20) fraction (previously described as a 3FTx and PLA_2_ respectively [10]), their lethal dose (LD_50_) were 5.9 µg/mouse and 0.85 µg/mouse, respectively. The electrophoresis evidenced molecular masses of 10 kDa and 14 kDa (Figure 2). These toxins were used in the immunization procedure, and the whole venom of *M. mipartitus* (Mm) was used.

**Figure 3:**
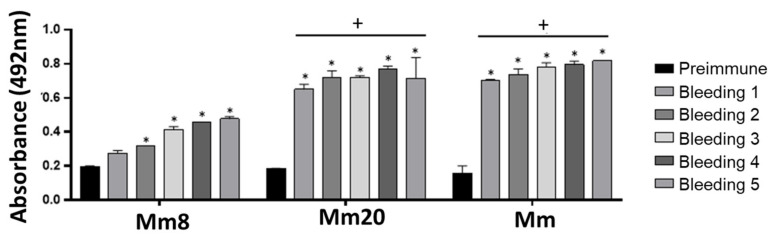
ELISA reactivity sera from each bleed against the *M. mipartitus* whole venom, Mm8 or Mm20. A 96-well plate was coated with each immunogen (Mm8, Mm20 and whole *M. mipartitus* venom). Serum from each bleeding was used at dilution of 1:100. * Indicates statistically significant differences with the preimmune serum. + indicates statistically significant differences against the Mm8 toxin (*p* < 0.05). Each bar represents the mean ± SD (*n* = 2).

**Figure 4:**
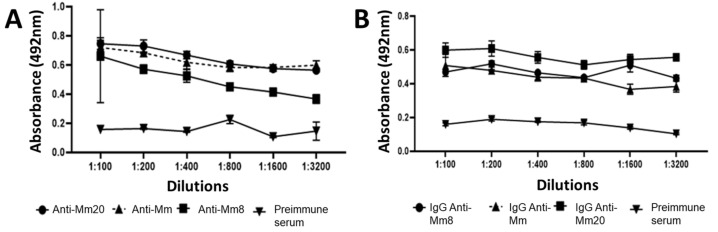
(**A**): Titration curve of antibodies in serum from bleeding five by ELISA against *M. mipartitus* whole venom and its fractions. A 96-well plate was coated with complete *M. mipartitus* venom and serum from bleeding five was used in dilutions from 1:100 to 1:3200. (**B**): Titration curve of each IgG by ELISA. A 96-well plate was coated with each immunogen (*M. mipartitus* whole venom, Mm8 and Mm20), and dilutions of each homologous IgG were added at 1:100 to 1:3200 dilutions. Bound antibodies were detected by a peroxidase-labeled anti-rabbit IgG conjugate. Each point represents the mean + SD (*n* = 2).

One rabbit for each toxin and venom was immunized during seven months using a scheme that started with a third of the LD_50_ of each immunogen (150 µg Mm8; 75 µg Mm20; 375 µg Mm) with an increase from 1.2 to 1.3 until the fifth immunization (472 µg; 236 µg, 787 µg, respectively) was deployed. In the last five boosters, the same dose was maintained. The serum samples were collected five times during the procedure and evaluated in their antibody levels against the isolated toxins and whole venom of *M. mipartitus*. Results showed that from the first immunization it was evident that there had been an increase in antibody titer. However, antibodies against the Mm8 toxin only had a significant increase after the third booster. In contrast, Mm20 and Mm induced an increase from the first immunization, in comparison with pre-immune serum (Figure 3). Further, the titers against them did not show significant differences between the booster shots. After last immunization (bleeding 5), all three serum showed titers up to 1:3200 (Figure 4A), and differences with pre-immune sera were significant even at low dilutions (1:100), especially with anti-Mm20 and anti-Mm.

### 2.2. Fractionation of Hyperimmune Serum and Titration IgGs

**Figure 5:**
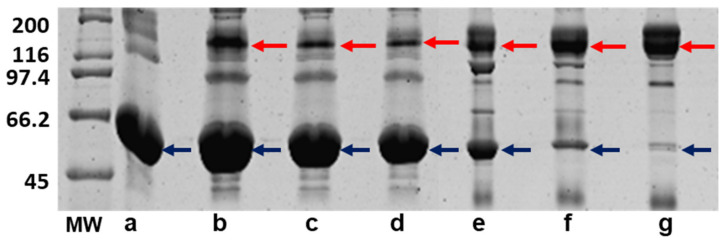
Hyperimmune sera and caprylic acid-extracted IgG were evaluated on 10% SDS-PAGE under non-reduced conditions and stained with Coomassie Blue R-250. MW: broad range molecular mass marker (kDa). (**a**): Albumin standard, (**b**): anti-Mm serum, (**c**): anti-Mm20 serum, (**d**): anti-Mm8 serum, (**e**): IgG obtained from anti-Mm serum, (**f**): IgG obtained from anti-Mm20 serum, (**g**): IgG obtained from anti-Mm8 serum. Red arrows indicate IgG band and blue arrows indicate albumin band.

The fractionation of each serum with caprylic acid facilitated the extraction of the IgG and reduction of other plasmatic proteins such as albumin, with an intensity increase of 150 kDa band in electrophoresis corresponding to IgG (Figure 5). The procedure efficiency was 1.6 mg/mL to anti-Mm8; 3.3 mg/mL to anti-Mm20 and 3.1 mg/mL to anti-Mm. 

Each IgG was prepared at a protein concentration of approximately 50 mg/mL and was tested against toxins and *M. mipartitus* venom by ELISA. The results showed reactivity of all IgGs against their own immunogens and titers up to 1:3200 were observed, but anti-Mm20 evidenced higher absorbance in the titers evaluated (Figure 4B).

**Figure 6:**
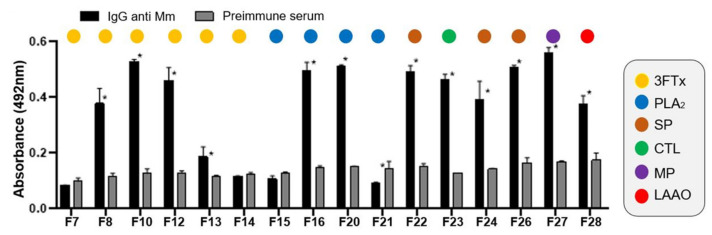
Cross-recognition of anti-toxin IgG against fractions obtained from *M. mipartitus* venom by RP-HPLC. Anti-Mm IgG recognition against protein families present in the whole venom according to the proteome described by [10]. Each bar represents the mean + SD (*n* = 2). * Indicates statistically significant differences with the preimmune serum.

**Figure 7:**
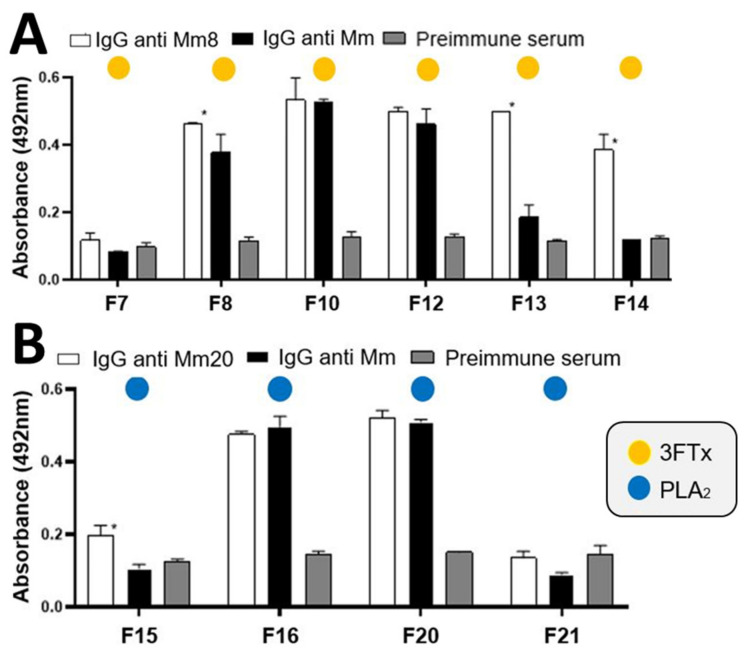
(**A**): Immunorecognition of anti-Mm8 and anti-Mm IgGs against fractions of the 3FTx family; * indicates statistically significant differences with respect to anti-Mm IgG. (**B**): Immunorecognition of anti-Mm20 and anti-Mm IgGs against fractions of the PLA2 family. Each bar represents the mean + SD (*n* = 2). * Indicates statistically significant differences with the preimmune serum.

IgG anti-Mm was evaluated in their cross reactivity against sixteen fractions obtained by RP-HPLC of *M. mipartitus* venom. The results showed a greater recognition of fractions 8, 10, 12, 16, 20, 22, 23, 24, 26, 27 and 28 (Figure 6). In the same way, a cross reaction between anti-Mm8 IgG and others 3FTx of venom was demonstrated (Figure 7A), it was even greater than that shown by anti-Mm IgG, with statistically significant differences for fraction 8 (*p* = 0.0192), fraction 13 (*p* < 0.0001) and fraction 14 (*p* < 0.0001). Likewise, the cross-reaction of anti-Mm20 IgG against other fractions of the venom corresponding to PLA2s (15 to 21; Figure 7B) was observed, and this was like the recognition shown by anti-Mm IgG against these same fractions, except for fraction 15, which was higher with anti-Mm20 IgG (*p* = 0.0005).

### 2.3. IgGs Neutralizing Ability against the Lethal Effect of M. Mipartitus

Each IgG (anti-Mm8 and anti-Mm20) was able to neutralize the lethal effect induced by its respective toxin (ED_50_: 1.5 mg toxin/mL). The anti-Mm ED_50_ was 1mg venom/mL. When the anti-Mm8 or anti-Mm20 were mixed individually with the whole venom, they did not neutralize its lethal effect. Nevertheless, when a mixture was made with 43% anti-Mm, 28.5% anti-Mm8 and 28.5% anti-Mm20, an increase in the neutralizing ability was observed compared with the anti-Mm alone. Additionally, a blend of anti-Mm8 and anti-Mm20 (ratio 1:2) was able to neutralize the whole venom.

## 3. Discussion

The development of new generations of antivenoms to treat snakebite accidents have focused particularly on different ways to fractionate the serum and to obtain the immunoglobulins or fragments to reduce the occurrence of adverse reactions. Whilst there have been variations in the methodology used for the immunization process, including different snake species from which the venom is obtained, and improvements in procedures used to obtain the IgG, the raw material used in the immunization continues to be the whole venom [14,15,16,17]. However, the availability of venom is a limiting factor in the production of antivenoms, especially for some species such as coral snakes, given the difficulties associated with maintaining them in captivity in relation to their diet and fossorial habits, and low venom production [18].

Similarly, the properties of snake venoms and their different levels of heterologous neutralization, have indicated that best approach to produce polyvalent antivenoms is to mix different monovalent antivenoms [19]. Nevertheless, some toxins of the venom are not neutralized successfully due to the low production of antibody titers in response to the low molecular mass of these toxins, in comparison with the high molecular mass components of the venoms [20]. It is important to note that low molecular mass toxins (3FTx and PLA_2_) are the most important in inducing the clinical manifestations in coral snakebites [6].

Proteomic has contributed to the discovery of venom components, which together with biological and biochemical tests has improved our understanding of medically important toxins [21,22]. Based on this information, several strategies have been proposed for the immunization process, such as the use of toxins mostly involved in envenoming and lethal effect, with the purpose of ensuring a greater amount of antibodies against those relevant toxins and, thus, an increase the clinical efficacy of the antivenom produced [23].

In this work, the venom of *M. mipartitus* was fractionated by HPLC and four toxins with a lethal effect on mice were obtained; these corresponded to two PLA_2_ and two 3FTx according to the proteome described by Rey-Suárez [10]. However, the LD_50_ found for Mm8 and Mm20 were 5.9 µg/mouse and 0.85 µg/mouse, respectively; these values differ from those reported by Rey-Suárez [10, 11] of 1.2 µg/mouse and 2 µg/mouse, respectively. This could be related to the use of different strains of mice (Swiss Webster in our study and CD1 in Rey-Suárez [10]) or the fact that a different batch of venom was used [24].

Considering Mm8 and Mm20 toxins are the most abundant and of clinical relevance in *M. mipartitus* envenomation, they were used here for the immunization process. They induced an immune response: the Mm8 toxin showed a lower response in the first immunizations in comparison with Mm20 or the whole venom, but after ten immunizations, its antibody titers increased. This may be related to the low immunogenicity of Mm8, which is a 3FTx with a molecular mass of 7029 kDa [11]. This observation was also reported by Fernández [25], who showed that 3FTx and PLA_2_ were the least recognized by an equine anticoral antivenom, indicating a lower response to these toxins during the equine immunization process. Laustsen [20] also induced a response to 3FTx using a murine model, but it was lower in relation to the response obtained with a PLA_2_ from *M. nigrocinctus* venom. In our work, it was observed the response obtained against PLA_2_ compared to 3FTx was greater from the first immunizations. The low immunogenic response to Mm8 was compensated by increasing its booster doses. On the other hand, antibody reactivity against PLA_2_ did not change after the fourth dose, which was similar to the response obtained when the whole venom was used.

Additionally in this work, it was found that anti-Mm8 IgG had more recognition of fractions of the 3FTx family than the showed by the anti-Mm IgG in the ELISA tests, suggesting a higher amount of antibodies against these toxins. Similarly, anti-Mm20 IgG also presented recognition to other fractions of the PLA_2_ family.

Some strategies using relevant toxins in the immunization process have demonstrated a better neutralizing capacity of sera. Chotwiwatthanakun [26] obtained a better result and an increase in the immune response to poorly immunogenic antigens, when mixtures of postsynaptic neuropeptides from elapids from Thailand were used for the immunization process, before the complete venom injections. Beghini [27] also showed a better neutralization of the toxic effects of the venom by immunizing both with the whole venom and with a PLA_2_ from *Crotalus durissus cascavella*. In the same way, Fusco [28] immunized rabbits with a PLA_2_ from *Crotalus durissus terrificus* prior to immunization with the whole venom. They found that 83.4 µg to 95 µg of IgG from serum of animals immunized with whole venom were needed to neutralize 1 µg of venom, but they needed only 54.9 µg of IgG from serum of animals immunized with whole venom with PLA_2_ to neutralize the same amount of venom. Other authors were able to produce a neutralizing coral antivenom, substituting *M. corallinus* by synthetic peptides derived from relevant toxins sequences [29].

We showed that a mixture of anti-Mm8 and anti-Mm20 neutralized the whole *M. mipartitus* venom. These results demonstrate the potential of these toxins to be produced by recombinant techniques and then be used as immunogens. This will allow the amount of venom necessary for the immunization process to be reduced, considering the limitations in the availability of venom from this species (*M. mipartitus*) whose maintenance in captivity is difficult.

Using isolated or recombinant expressed neurotoxins from several species of coral snake for immunization might represent a strategy to produce a therapeutically enhanced antivenom with cross- reactivity against several *Micrurus* venom toxins to reduce the reactivity against medically irrelevant toxins.

## 4. Conclusions

The results of this work demonstrated that it is possible to produce antibodies against the main toxins responsible for the toxicity of *M. mipartitus* venom, thus improving the representativeness of the antibodies against these toxins and generating a greater neutralizing capacity of the antivenoms against this venom. Additionally, having demonstrated that the antibodies produced against these toxins neutralize the complete venom, it is suggested that there is a possibility to develop and use recombinant 3FTx and PLA_2_ toxins as immunogens to produce antivenoms. This is thought to represent an alternative to reducing the amount of venom used in anti-coral antivenom production.

## 5. Materials and Methods

### 5.1. Venoms and Animals

*M. mipartitus* venom was provided by the serpentarium of the University of Antioquia and obtained from adult specimens collected in the southwestern region of Antioquia. This venom was pooled, lyophilized, and stored at −20 °C.

Female and male Swiss Webster mice (18–20 g) and New Zealand female rabbits (2 kg) were used and supplied by the Animal House (Sede de Investigacion Universitaria–SIU) of the Universidad de Antioquia. All animals received food and water ad libitum under controlled environmental conditions. The experimental protocol of the Project CODI-UdeA Nro 764/2018 was approved by the institutional Committee for the Use and Care of Research Animals at the Universidad de Antioquia, license nos122 (13 February 2019).

### 5.2. Isolation of Toxins from M. mipartitus Venom

Reverse-phase (RP) HPLC was performed following the protocol described by Rey-Suárez [10]. Two milligrams of venom were dissolved in 200 μL of water containing 0.1% trifluoroacetic acid (TFA; solution A), centrifuged for 5 min at 4500 rpm, and applied to a C18 column (250 × 4.6 mm, 5 µm particle; Restek, Bellefonte, PA, USA) using a Shimadzu Priminence-20A chromatograph (Columbia, SC, USA). Elution was performed at 1 mL/min by applying solution B (acetonitrile, containing 0.1% TFA) as follows: 5–15% B for 10 min, 15–45% B over 60 min, 45–70% over 12 min. The elution profile was monitored at 215 nm in a UV/VIS photodiode array detector (Shimadzu, Kyoto, Japan). Fractions were manually collected and dried in a centrifuge Eppendorf Vacufuge plus (Hamburgo, Germany). The fractions were redissolved in distilled water and their protein concentration was quantified in a Nanodrop 2000 from Thermo Scientific (Waltham, MA, USA).

The most abundant fractions were screened for lethal effect (using a dose of 5 μg/mice). The LD_50_ of the lethal fractions was determined by injecting different doses (0, 5–10 μg) in 300 μL of PBS by the intraperitoneal (i.p.) route, in groups of three mice. A control group received PBS alone. Mice were monitored for 24 h and LD_50_ was calculated with Probits [30,31]. The fractions with the lowest LD_50_ and the highest abundance were selected for the immunization processes.

Furthermore, the peaks were evaluated by SDS-PAGE. For this, 20 µg of protein was loaded into a 12% polyacrylamide gel and run in a Mini-Protean Tetra^®^ electrophoresis system (Bio-Rad, Hercules, CA, USA) at 150 V. The gel was subsequently stained with Coomassie blue R-250 [32].

Finally, the homogeneity of the fractions used in immunizations was verified by RP-HPLC; 50 or 100 μg of each toxin were injected in the same chromatographic system using a gradient from 0 to 70% of B solution.

### 5.3. Immunization Protocol

Adult New Zealand rabbits were injected subcutaneously with each toxin selected or the complete *M. mipartitus* venom, combined with incomplete Freund’s adjuvant. After collection of non-immune sera, animals received an initial dose and, every 3 weeks, booster injections were made using the same conditions. During the process and one week after the last immunization, the rabbits were bled to obtain serum and evaluate the antibody titer. The sera obtained were frozen at −20 °C.

### 5.4. IgG Purification

IgGs were purified from immunized rabbits’ sera. The IgGs were concentrated using caprylic acid as described by Steinbuch & Audran [33]. Small amounts of caprylic acid (Sigma, Saint Louis, MO, USA) were added to each serum until they reached 5% of the total volume. The supernatant was dialyzed on Fisherbrand cellulose membranes (3500 mwco) with PBS and distilled water. Finally, it was lyophilized and stored at −20 °C.

### 5.5. Antibody Titers

After each test and final bleeding, an ELISA was performed to determine and quantify antibody production, following the method described by Lomonte [34]. A 96-well plate (Falcon ref 35072) was covered with 0.1 µg/well of complete *M. mipartitus* venom or of each toxin. Serum and IgG dilutions were added: 1:100 for the test bleeds and up to 1:3200 for the final bleed, diluted in a blocking buffer. The absorbances were read at 492 nm in a Multiskan sky spectrophotometer from Thermo Scientific (Waltham, MA, USA). Preimmune serum was used as a negative control in the same dilutions. Each sample was analyzed in duplicate.

The cross-reactivity of the IgGs against the fractions corresponding to 3FTx and PLA_2_ of the *M. mipartitus* venom obtained in RP-HPLC according to Rey-Suárez [10] was evaluated by ELISA; for this, the plate was covered with 0.1 µg of each fraction and, following the protocol previously described, a 1:400 dilution of each IgG was used. Pre-immune serum was used as a negative control.

### 5.6. Neutralization of the Lethal Effect of Venom and Toxins with IgGs

The lethality neutralizing capacity of either IgGs was determined by blending 1.5 LD_50_ of the whole venom or 3FTx or 2LD_50_ of PLA_2_, with different doses of its homologous IgG, incubated at 37 °C for 30 min and injected intraperitoneally into groups of three mice. The mice were observed for a period of 24 h, and the Effective Dose 50 (ED_50_) was determined by probits. A group of mice injected only with the venom was used as a positive control [15,31].

Likewise, to determine if the antitoxins were able to neutralize the lethal effect of the whole venom, variable doses of both antitoxins were incubated with 1.5 LD_50_ of the venom; the mixture was injected into groups of three mice. Finally, to determine if the complete antivenom IgG (antiMm IgG) improved its neutralizing capacity when combined with antitoxin IgGs, the lowest dose of antiMm IgG was used and mixed with variable doses of both antitoxin and injected into groups of three mice.

### 5.7. Statistical Analysis

Results were expressed as mean ± SD and they were analyzed using GraphPad Prism 8.0.1. Normality was evaluated using a Shapiro–Wilk test; analysis was performed with two-way ANOVA followed by a Bonferroni test for multiple comparisons. They were statistically significant when *p* < 0.05.

## Data Availability

Not applicable.
